# General Practitioners (GP) experiences in managing patients in clinical trials conducted by non-GP specialists: An exploratory qualitative study

**DOI:** 10.1017/cts.2025.10217

**Published:** 2025-12-19

**Authors:** Tran Ngoc-Bao Lam, Yasin Shahab, Phyllis Lau

**Affiliations:** 1 Royal Australia College of General Practitioners, Department of General Practice, School of Medicine, Western Sydney University, Penrith, New South Wales, Australia; 2 Department of General Practice, School of Medicine, Western Sydney University, Penrith, New South Wales, Australia

**Keywords:** General practitioners (GPs), primary care physicians (PCPs), primary care research/trials, communication, non-GP specialists

## Abstract

**Introduction:**

General Practitioners (GPs) in Australia continue to provide primary medical care for patients enrolled in specialist-led clinical trials (CTs), yet research on GPs’ experiences in this role remains limited. This study explored Australian GPs’ experiences managing CT patients and their perspectives on primary care involvement in clinical research.

**Methods:**

This phenomenological study involved 11 semi-structured interviews with GPs in New South Wales, Australia, between July to October 2024, who previously managed patients enrolled in other non-GP subspecialist CTs. The recruitment applied purposive, snowballing and convenience sampling approaches. Interviews were transcribed, inductively coded and thematically analyzed.

**Results:**

Analysis revealed four themes: (i) lack of communication from CT teams, (ii) patients’ insufficient understanding about their CTs, (iii) time and resource barriers to GP involvement in CTs, (iv) varied opinions about GPs playing an active role as researchers in CTs.

**Conclusions:**

This exploratory study highlighted the lack of proper communication gaps between CT teams and GPs, potentially compromising the quality of patient care. Digital health records (such as Australia’s My Health Record) could facilitate information sharing. While GPs support primary care research, barriers include limited research training, time constraints, and inadequate resources. Integrating research education into GP training and establishing practice-based research networks could enhance GP participation in CTs.

## Introduction

Although the majority of patients enrolled in clinical trials (CTs) in Australia are monitored and followed up by specialized clinics or non-GP specialists who are responsible for their enrollment, General Practitioners (GPs) continue to be the patient’s primary contact for general health advice and management of their other comorbidities [1]. In Australia, GPs are the first service people go to for health care, provide diagnosis and treatment of a wide range of health conditions and injuries, as well as long-term care [2]. Previous studies have explored the role of primary care physicians/GPs in the designs of CTs to better monitor the progress of their patients throughout the trial duration. Researchers from various countries have considered engaging GPs and their practice staff in CTs so that the end results would be more applicable at the community level compared with results of CTs organized solely at the hospital or tertiary care level [3]. Armstrong et al. from Australia emphasized the importance of providing comprehensive information about CTs to GPs and the role of GPs in designing clinical studies for cancer treatment [4]. Greater coordination among primary care physicians (PCPs) and cancer centers has been shown to improve patient trust in oncologists and subsequently facilitate trial enrollment [5].

While there might be differences in the health system in other countries (for example in Germany where GPs do not act as gatekeepers to specialized care and compete with other physicians in the community for patients), the feasibility of CTs in primary care and the common barriers that prevent GPs from participating in CTs are of special interest [6,7]. Bylund et al. found that despite having little experience and low knowledge level about trials, PCPs in England had positive attitudes and beliefs about these studies [8]. The authors recognized that PCPs are likely to help improve patients’ participation in cancer CTs given the trust their patients place in them [8]. Millar et al. in the US noted that CT recruitment in the primary care setting is an ongoing challenge [9]. Taft et al. reported deep concerns from PCPs in the US regarding their loss of control in protecting patient’s wellbeing and managing clinic workflow when their patients participate in CTs. They suggested creating a standard support system and processes to facilitate quick access of CT information for PCPs [10].

Despite the role of GPs in recruiting and managing CT patients being increasingly recognized, a 2017 cross-sectional study conducted by a Spanish hospital found that while more than two-third of their CT protocols acknowledged the role of PCPs, only 6% documented the method of communication with GPs [1]. In Australia, there have been a few studies that highlighted the role of GPs in patients’ recruitment into CTs [3]. There is little research on the experience of GPs managing CT patients, specifically when community-based health providers such as GPs are not part of the trial protocols.

Our exploratory study explored the experience of GPs who have cared or are caring for patients enrolled in specialized clinic or hospital-based CTs, examined their role and their views about engaging more actively as researchers in CTs. We hope the findings could inspire larger-scale research to inform the future designs of non-GP specialist led CT protocols supporting GPs in their management of patients in the community and ensuring patient’s safety during and after CTs. This study may also provide further evidence to support the active involvement of GPs as CT researchers.

## Methods

### Study design

An exploratory qualitative study using individual semi-structured interviews was conducted to explore the experience and views of GPs who had or have patients enrolled in specialized clinic or hospital-based CTs. The Western Sydney University Ethics Committee granted the study HREC Approval Number H15968 on 21/05/2024 as it meets the requirements of the National Statement on Ethical Conduct in Human Research 2023.

### Participants and recruitment

Eligible participants included GPs currently practicing in Australia and have either cared or are caring for patients enrolled in specialized clinic or hospital-based CTs. A purposive and convenience approach was used initially, then a snowball approach for selection and recruitment through the research team’s professional networks and Practice-Based Research Networks (PBRNs). Over 150 GPs were first contacted by TL via email or telephone, and, if they indicated an interest in participating, a Participant Information Sheet and basic demographic information questionnaire were provided before a consent form was signed and returned to us electronically. The questionnaire contained questions on age range, gender, practice location, practice type, years of professional experience, and whether they have cared for and are caring for patients participating in CTs. The survey used for demographic data has not been previously validated and was developed for this study to screen participants to ensure participant eligibility and obtain a broad representation of perspectives. The survey was anonymous to protect participant privacy. Participants were then invited to attend individual semi-structured video teleconference interviews estimated to last between 20 and 30 minutes.

### Data collection

An interview guide was developed by the research team based on review of the literature. It consisted of questions on participants’ experience of caring for patients enrolled in specialized clinic or hospital-based CTs, their views about their current roles and playing a more active role as researchers in CTs (Table 1). Pilot interviews were conducted with 2 academic GPs in the Department of General Practice at Western Sydney University to refine the comprehensiveness and clarity of the questions.

**Table 1.** Interview questions

1. I understand that you have cared for patients on clinical trials. Can you please tell me more about it? Probe: – What sort of interventional trial were they enrolled in? Please give examples. – How long had your patients been on those trials? Please give examples.
2. What are some challenges that you may have when managing a patient enrolled in a clinical trial? (Prompts: drug interactions with usual meds)
3. How do you usually find out about your patient ‘s participation in clinical trials? (Prompts: patients told you, patients asked your opinions about enrolling in a trial, clinical trial coordinator contacted you, you actually enrolled the patient, etc.)
4. If you have ever had communication with clinical trial coordinators, what is your opinion about the level or quality of the communication? Probe: – If you were not satisfied with the level of communication, what would you suggest to improve it?
5. In your opinion, what is the best way to inform GPs that their patients are enrolled in a clinical trial? (Prompts: via phone, email, fax, zoom, mail etc.) Probe: Some people have suggested to register patient’s enrolment in clinical trial in My Health Record What are your thoughts about that?
6. I see you have indicated previously that you were/were not actually involved in clinical trials (e.g. as CI, AI, recruiting patients, collecting data). Please tell me your experiences. Probes: (If they have been involved before) Would you be involved similarly again? (If they have not been involved before) Would you like to be more involved? How? (If they have never been involved as a CI or AI) If the opportunity arises, would you be interested to be an investigator on a clinical trial?

CI = Chief Investigator; AI = Associate Investigator.

Individual semi-structured interviews were conducted by TL with participants via videoconference, and audio-recorded. The recordings were transcribed verbatim. The transcripts were offered to all participants and provided upon request. One GP requested, reviewed, and confirmed the accuracy of their interview transcript. The recruitment and interviews continued until data sufficiency was determined to have been achieved by the research team, i.e., when the “dataset is comprehensive enough (depth) to both identify recurrent thematic patterns and to account for discrepant examples (breadth)” [11,12]. A fifty-dollar gift card was provided to participating GPs as compensation for their time.

### Data analysis

The interview transcripts were then organized and coded using the NVivo software [13]. Data collection and analysis occurred concurrently. An inductive thematic analysis approach was used for identifying, analyzing and interpreting the themes within the qualitative data to create the report including a selection of illustrative quotes from participants [14].

All data were coded by researcher TL. YS separately coded two randomly selected transcripts and both TL and YS cross-checked their coding to compare understanding of the data. Any discrepancies in their interpretations were discussed with PL to resolve differences. Themes and subthemes were then generated by TL by identifying patterns and grouping and integrating similar codes. Consensus on the data analysis was reached through regular research team meetings to ensure all perspectives were considered, consistency in coding and theme development.

## Results

From July to October 2024, 11 eligible GPs currently practicing in New South Wales, Australia were recruited and interviewed. There were six females and five male participants with a wide range of years of professional experience. Three had up to 40 years of practicing as a GP. Most worked in metropolitan areas of Sydney in private practice (Table 2). Each interview lasted an average of 20 minutes. Data sufficiency was determined to have been reached by the research team.

**Table 2.** GPs demographics

	Participant gender		Years of professional experience					Type of practice			Practice location	
	Female	Male	0–5 years	11–15 years	16–20 years	21–30 years	31–40 years	Private	Community based (Aboriginal)	Community based (Refugee Health service)	Metropolitan	Rural
Number of GPs	6	5	3	3	1	1	3	8	2	1	8	3

*General Practitioners = Gps.*

Community-based Aboriginal health services = specialty primary care services for the Indigenous population of Australia.

Community based (Refugee Health service) = specialty primary care services for the Refugee population of Australia.

There were four themes elicited from the interview data (Table 3): (i) lack of communication from CT teams, (ii) patients need more information about their CTs, (iii) challenges of clinical trials in primary care, (iv) varied opinions about GPs playing an active role as researchers in CTs.

**Table 3.** Summary of responses

Participant	Background information	Summary of responses			
		Lack of communication from CT teams?	Patients need more information about their CT?	Mentioning challenges of CTs in primary care	Should GPs play an active role as researchers in CTs?
1	Male, 61–70 years old, 21–30 years of professional experience, private metropolitan practice	Yes	Yes	Yes	Unsure given the challenges
2	Female, 18–30 years old, 0–5 years of professional experience, private metropolitan practice	Yes	Yes	Yes	Supported
3	Female, 41–50 years old, 16–20 years of professional experience, Aboriginal health rural practice	Yes	The participant searched for CTs and introduced her patient to them	Yes	Supported
4	Male, 41–50 years old, 11–15 years of professional experience, private rural practice	Yes	No – patients have got information from the CT team	Yes	“I’m not super into research”
5	Male, 61–70 years old, 31–40 years of professional experience, private rural practice	Yes	No	Yes	Supported
6	Female, 31–40 years old, 11–15 years of professional experience, private metropolitan practice	Yes	No	Yes	Supported
7	Female, 31–40 years old, 11–15 years of professional experience, Refugee health metropolitan practice	Yes	Yes	Yes	“I would say. GPs are probably more expert at doing a trial on something involved in general practice” “But we are general practitioners, we are generalists … I don’t actually know what the rules are. Non specialists are not allowed to run trials but I would think GPs are allowed to run trials.”
8	Male, 18–30 years old, 0–5 years of professional experience, private metropolitan practice	Yes	Yes	Yes	“Unlikely”
9	Female, 61–70 years old, 31–40 years of professional experience, Aboriginal health metropolitan practice	No	No	Yes	“I am willing to be a part of that”
10	Female, 18–30 years old, 0–5 years of professional experience, private metropolitan practice	Unsure – “They (CT team) sent some document, but I cannot remember”	Unsure	Yes	Supported
11	Male, 61–70 years old, 31–40 years of professional experience, private metropolitan practice	No	No	No	Supported

*Clinical trial = CT; General Practitioners = GPs.*

*Community-based Aboriginal health services = specialty primary care services for the Indigenous population of Australia.*

*Community based (Refugee Health service) = specialty primary care services for the Refugee population of Australia.*

### Lack of communication from clinical trial (CT) teams

Many GPs reported that they had limited communication from CT teams about the trials that their patients were taking part in.

“They all just happened without having talked to me about it first.” *(Participant 1)*

“I think that was about six to eight weeks after the patient started taking medication from the trial until I received the first letter from the specialist.” *(Participant 2)*

Even when GPs were informed, the information from the CT team was inadequate.

“When she (the patient) had actually enrolled on the trial formally, the specialist letter did inform me, but not with any further details of the trial, just as a part of the communication after their appointment…I guess…they would just assume that they’re following that up and that maybe we didn’t need to know.” *(Participant 3)*

“The local specialist that I referred to wrote back to me, saying “…, I’m gonna refer them to…the trial”… They certainly don’t write back to me with any details about what the trial is, … what’s going on. They’re sort of handing it off to someone else, and I would assume that they would think if they wanted me to know more, that specialist would get in contact with me, or that trial group…You’re like a third wheel with it. I was not really involved with it at all. Usually, I only find out from my patients that they’re on a trial…” *(Participant 4)*

CT teams sometimes contacted GPs but only when they need GPs to confirm any changes in patients’ medication or to help order and follow up further investigations.

“There’s probably only one trial that I actually received some information. And it was basically around the fact that they just wanted to know if I was changing any of their medication.” *(Participant 4)*

Most GPs recognized that the poor communication between GPs, and the CT team potentially posed risks to patient safety.

“It’s not a drug I’m going to be familiar with. I don’t know how it interacts with anything else. And that’s the first time I hear about it. So, I don’t get any warning or any background information about it. So that’s a bit of a problem” *(Participant 1)*

“I had to send off the results and things to one person from the trial where the patient’s just on a treatment but I have no idea who the trial coordinator is or whose investigators are or I don’t even know what the research question is.” *(Participant 5)*

In order to improve communication between GPs and CT teams, some participant suggested utilizing the My Health Record platform (which is an Australian government digital electronic health record accessible by clinicians and patients [15]. The inclusion of the patient’s GP was suggested as a requirement for enrolling in a CT.

“It would be good if clinical trial info can be included in My Health Record, to create a new icon and new column for the clinical trial. At the software that there’s a new icon reminding us on the clinical trial, because the clinical trial medication is not usually reflective on our medication list.” *(Participant 2)*

“… for the communication between GPs and the clinical trial teams, in order to ensure proper care and the safety of the patient throughout, and then, at the end of the trials, I would wonder if it’s actually worth it if patient is getting enrolled into a study only if they can provide the contact details of their GP.” *(Participant 6)*

### Patients’ insufficient understanding about their clinical trials

Some participants felt that some of their patients did not know much about the trial or the trial medications they were receiving.

“I doubt how many of those patients understand that information and I am not investigating how much they understand because I don’t understand myself.” *(Participant 1)*

Participants thought that GPs was a source of information for these patients.

“We always need to check with patients, because sometimes patients might not understand like how the regimen was proposed by the specialist and the GP need to seek information more often, need to correct them, or remind them, or help them, or answer their concerns.” *(Participant 2)*

“I think one patient came back to me to ask a little bit more information after they read about it (the trial) in more detail. We went through those questions.” *(Participant 7)*

### Time and resource barriers to GP involvement in Clinical Trials

Participants talked about the dearth of CTs conducted in the primary care setting despite the high patient volume.

“We are working in primary care with much higher number of patients, compared with the patient from hospital or specialists at the same time. But I don’t think that we have a lot of trials and research on.” *(Participant 6)*

Some participants, nevertheless, expressed concerns about the lack of GP input in CTs even when primary care was directly involved.

“I also do wonder when it comes to clinical trials. A lot of the time, the GPs’ perspective is missing. You’ll have trials where they have a big panel of various doctors and they’ll all be sub-specialists and there is no GP, even if those trials directly affect us in general.” *(Participant 6)*

“A lot of research would be more useful if it included a primary care setting. A lot of research that we have to use in primary care is primarily generated in hospital setting and its applicability to the primary care setting is possibly different.” *(Participant 5)*

Some participants were candid about the time and financial constraints for GPs to be actively conducting CTs.

“Because general practice is a private entity as opposed to a clinical trial in a hospital where the specialist is getting their wage, I think another big barrier in general practice is just time to do it and I think cost would be a factor as well.” *(Participant 3)*

“If you want to look into an academic career, I guess our guidelines always need updating and it takes so long for research to go into guidelines. I guess you would lose that on income if you take the time to do trials and things.” *(Participant 1)*

### Varied opinions about GPs playing an active role as researchers in clinical trials

When asked whether they would consider actively leading CTs in primary care, most were not interested, for personal reasons.

“Personally, I’m not super into research. And we’re so busy with other stuff…I usually just trying to keep my head above water with work as it is.” *(Participant 4)*

“Unlikely. Do I have to stay back more? Do I have to come in early? Do I have to book out a whole day, so I don’t know.” *(Participant 8)*

Some, however, felt the importance of distinction between community and hospital trials, and would be willing to play an active role as researchers in primary care CTs.

“If it’s something very relevant to general practice, I don’t see why they (GPs) couldn’t run a trial. I would say. GPs are probably more expert at doing a trial on something involved in general practice more than, say, a cardiologist getting involved in that trial because a cardiologist is more focused on cardiology.” *(Participant 7)*

“I would hope that GPs these days would realise how important it is to know about the impact of medications, or whatever’s being investigated on a patient in the community as opposed to a patient in hospital and would be aware of the importance of patients in the community being involved in clinical trials. Therefore, I am willing to be a part of that.” *(Participant 9)*

Some participants specifically mentioned the perception that other specialty disciplines have of GPs not being capable or interested to conduct CTs.

“I have gone through hospital medicine. They all think of us as not specialists, so it wouldn’t surprise you that everyone else would hold that bias as well… They might even think that we’re not interested. Or that we don’t need to know, but I do think it probably does contribute to the gap.” *(Participant 9)*

Some also revealed feelings of uncertainty about GP’s capacity to conduct CTs.

“What’s the definition of a specialist? So you know, we call ourselves specialists in life. But we are general practitioners, we are generalists. Specialists usually specialise in a particular area. So I mean, I don’t actually know what the rules are. Non specialists are not allowed to run trials but I would think GPs are allowed to run trials.” *(Participant 7)*

## Discussion

Clinical trials (CTs) in medicine are continuously emerging and have become a critical component in the development of new medical treatments and interventions aimed at improving healthcare quality. However, Crowley W et al. described clinical research as “a fragmented cottage industry constituted of multiple stakeholders… with no overarching vision” [16]. In Australia, GPs play a pivotal gatekeeper role within the health care system, yet their level of involvement in medical research varies. Whilst many CTs primarily involve non-GP specialists or research institutions, GPs are often less engaged. This study hopes to explore GPs’ experience in managing patients participating in CTs run by non-GP specialists and to assess their perspectives about CTs in the primary care sector.

Our findings highlight a significant communication gap between CT teams and GPs when their patients are involved in CTs, leading to concerns about the potential for unavoidable errors by GPs and the subsequent impact on patient safety. Several of our participants felt that patients were often insufficiently informed about the trials they were involved in and emphasized the importance of GPs providing further assistance and guidance. When discussing practical solutions, GPs proposed using the digital health record, My Health Record, and incorporating the GP’s details in CT patient enrollment to improve coordination and communication between GPs and the CT teams. Time and financial constraints are the primary factors contributing to the lack of interest among most GPs in leading CTs in primary care. Some participants expressed uncertainty about their capacity to conduct CTs. They also reflected on their assumption that non-GP specialist colleagues believe GPs lack both the interest and the expertise to lead CTs. Nonetheless, our participants remain optimistic about the future of primary care CTs and are open to playing a research active role.

### Communication challenge

Our findings indicate that GPs are concerned about the communication gap with hospital CT team and the lack of opportunity to have their voice heard in a multidisciplinary team. Antoni Schoenenberger-Arnaiz, et al found that the majority of PCPs were not notified when their patients enrolled in a trial at a Spanish hospital-based research institute even though the trial protocol specifically included a requirement for the CT investigators to inform family physicians [1]. There was also an expectation that the patients would notify their family physicians instead [1]. Similarly, GPs and primary care seemed to be missing from the picture of multidisciplinary care for cancer CT patients in the study by Wen-Ying et al in a US teaching hospital [17]. The study highlights the inherently hierarchical nature of hospitals, where decisions made by the CT research team members took precedence over the priorities of other healthcare professionals, ultimately affecting the continuity of care and the best interests of the patient [17]. It is critical to address the gap in communication with GPs and involvement of GPs in CTs research if patients enrolled in CTs by hospitals or research institutes continue to be followed up by GPs for management of comorbidities, toxicities, or informational support [1].

There is evidence in the literature that the general public’s understanding of clinical research is considerably limited [1,18]. The role of GPs as providers of clinical research information was emphasized by our GP participants, a view supported by patients in the 2013, 2015, and 2017 Center for Information and Study on Clinical Research Participation (CISCRP) studies in the US, who consistently preferred to receive information about clinical research from their regular physicians or specialists rather than any other source [19]. Most CT patients interviewed by Antoni Schoenenberger-Arnaiz et al. wanted their family doctors to be informed about their participation in trials [1]. It was noted that only 15% of the patients in this study could recall the name of the trials they were involved in [1].

Zhou et al. highlighted the importance of implementing strategies to enhance interdisciplinary communication in order to improve patient recruitment and retention in CTs [20]. In addition to traditional methods such as correspondence letters and clinical study ID cards for patient participants, a 2017 study in Spain recommended using the health system’s electronic records with an alert feature to notify when the patient enrolled in a CT [1]. This could act as a platform for sharing trial information between non-GP specialists and PCPs [1]. Most interviewees in our study also supported the inclusion of CT information in patients’ My Health Record.

### Primary clinical trials led by GPs

According to the US Institute of Medicine, family physicians are less likely to incorporate research findings from academic medical centers into their daily practice compared to those conducted in community settings [21]. This underscores the crucial role of primary health research and trials. Still, our GP participants expressed their concern about the practical relevance of findings from so-called primary care CTs when their input was not sought. On the other hand, primary care participation in clinical research, especially randomized controlled CTs, continue to face significant challenges in recruiting and maintaining a sufficient number of GPs for research [22]. Researchers in German and US share the same conclusion that busy patient practice, financial pressure from reducing clinical consultations for research activities, and lack of supportive research infrastructure, education and network are the main barriers for GP’s involvement in clinical research and trials [21,22]. These factors were also major concerns for our GP interviewees when considering more active participation in CTs. It indicates that the current primary care setting is not well-aligned with the significant growth and demand of clinical research. In Australia, GP clinics usually operates as small private businesses and do not have the resources of established research centers. It was noted that GPs received little research education during their specialization training compared to other specialists [23]. Unlike their colleagues from other specialties when research is integral to the hospital environment, GPs’ access to research/CT information and opportunities is limited [23]. In a survey completed by thirty-four GP participants enrolled in the LEAP study, a randomized controlled trial aimed at reducing childhood overweight conducted in primary care settings in Melbourne, Australia, most GPs expressed their concern about their role in facilitating the research at the general practice level. The majority of them had no prior training in research methods, and none held a higher degree in research at the Masters level or above [24]. This may help explain the assumption held by some of our GP participants and non-GP specialist colleagues, that GPs are not adequately prepared to lead CTs. Given that many GP participants in the LEAP study expressed a desire to gain knowledge and clinical skills that could be applied to their daily practice, future researchers were encouraged to clearly outline the clinical benefits of their studies for both GPs and patients in order to increase GP engagement [24]. This is congruent with our interviewees’ support for their involvement in primary care CTs that are relevant to general practice. The introduction of research networks in primary health care services has been successfully engaging more GPs contribution in Europe and North America [1]. Likewise, PBRNs have been established since early 2000s in Australia, despite lacking sustainable funding, to engage with and build research capacity of GPs and other primary healthcare providers [25].

### Limitations of the study

The limitation was the difficulty in recruiting a wide breadth of GPs, which may have impacted the diversity of opinions obtained. There was lack of awareness among GPs regarding their patients’ participation in CT due to the absence of an alert system in Australian general practice software at the time of data collection. Our participants were either informed by their patients or CT teams. This study explored the experiences and perspectives of GPs. Future research should include the experiences and perspectives of CT coordinators and other specialist researchers in managing trial patients within the community and communicating with their GPs.

## Conclusion

Information exchange between GPs and CT teams is limited which can impact on patient’s knowledge about CTs and the quality of care. The health system’s electronic records such as My Health Record can be an effective platform for sharing CT information between the trial team and primary care providers. Primary care CTs has the potential to play a crucial role in shaping the future of general practice, yet GPs face a number of challenges in engaging and contributing as CT researchers. To support their participation, there is a need to integrate research education into GP training program and establish connections through PBRNs.

## References

[1] Antoni Schoenenberger-Arnaiz JA , Solanilla-Puertolas M , Acer-Puig M , Gomez-Arbones J. Informing primary care physicians of patients’ involvement in clinical trials carried out at a specialist care level. Open Access J Clin Trials. 2017;9:59–64. doi: 10.2147/OAJCT.S134555.

[2] Health. Australian Government Department of Health, Disability and Ageing. 2025, (https://www.health.gov.au/primary-health-care-careers/general-practitioners?language=en) Accessed January 7, 2025.

[3] Nelson MR. General practice-based clinical trials. MJA. 2013;198:136–137. doi: 10.5694/mja12.11061. 23418685

[4] Armstrong Ruth M , Mabel C , Martin VDW. The go-between: general practitioners and clinical trials. Med J Aust. 1999;171:301–302. doi: 10.5694/j.1326-5377.1999.tb123660.x. 10560445

[5] Sprague Martinez L , Freeman ER , Winkfield KM. Perceptions of cancer care and clinical trials in the black community: implications for care coordination between oncology and primary care teams. J Oncol. 2017;22:1094–1101. 10.1634/theoncologist.2017-0122 PMC5599206 28706009

[6] Gágyor I , Bleidorn J , Wegscheider K , Hummers-Pradier E , Kochen MM. Practices, patients and (im)perfect data – feasibility of a randomised controlled clinical drug trial in German general practices. Trials. 2011;12:91–91. doi: 10.1186/1745-6215-12-91. 21457558 PMC3080301

[7] Hummers-Pradier E , Bleidorn J , Schmiemann G , et al. General practice-based clinical trials in Germany – a problem analysis. Trials. 2012;13:205–205. doi: 10.1186/1745-6215-13-205. 23136890 PMC3543296

[8] Bylund CL , Weiss ES , Michaels M , et al. Primary care physicians’ attitudes and beliefs about cancer clinical trials. Clin Trials. 2017;14:518–525. doi: 10.1177/1740774517717722. 28693389 PMC5663299

[9] Millar MM , Taft T , Weir CR. Clinical trial recruitment in primary care: exploratory factor analysis of a questionnaire to measure barriers and facilitators to primary care providers’ involvement. BMC Prim Care. 2022;23:311. doi: 10.1186/s12875-022-01898-2. 36463123 PMC9719201

[10] Taft T , Weir C , Kramer H , Facelli JC. Primary care perspectives on implementation of clinical trial recruitment. J Clin Transl Sci. 2019;4:61–68. doi: 10.1017/cts.2019.435. 32257412 PMC7103461

[11] Braun V , Clarke V. To saturate or not to saturate? Questioning data saturation as a useful concept for thematic analysis and sample-size rationales. Qual Res Sport Exerc Health. 2021;13:201–216. doi: 10.1080/2159676X.2019.1704846.

[12] LaDonna KA , Artino AR Jr , Balmer DF. Beyond the guise of saturation: rigor and qualitative interview data. J Grad Med Educ. 2021;13:607–611. doi: 10.4300/JGME-D-21-00752.1. 34721785 PMC8527935

[13] Houghton C , Murphy K , Meehan B , et al. From screening to synthesis: using nvivo to enhance transparency in qualitative evidence synthesis. J Clin Nurs. 2017;26:873–881. doi: 10.1111/jocn.13443. 27324875

[14] Braun V , Clarke V. Using thematic analysis in psychology. Qual Res Psychol. 2006;3:77–101. doi: 10.1191/1478088706qp063oa.

[15] Australian Digital Health Agency. My health record [Internet]. Australian Digital Health Agency. 2025, (https://www.digitalhealth.gov.au/initiatives-and-programs/my-health-record) Accessed January 7, 2025.

[16] Crowley W , Sherwood L , Salber P , et al. Clinical research in the United States at a crossroads. Proposal for a novel public-private partnership to establish a National clinical research enterprise. JAMA. 2004;291:1120–1126. doi: 10.1001/jama.291.9.1120. 14996782

[17] Wen-Ying SC , Angela LF , Kathleen C , et al. Cancer clinical trial providers’ perspectives on communicating goals of care: a key informant study. PEC Innovation. 2022;1:100041–100041. doi: 10.1016/j.pecinn.2022.100041. 37213723 PMC10194320

[18] Anderson A , Borfitz D , Getz K. Global public attitudes about clinical research and patient experiences with clinical trials. JAMA Netw Open. 2018;1:e182969. doi: 10.1001/jamanetworkopen.2018.2969. 30646218 PMC6324429

[19] Getz KA. Examining and enabling the role of health care providers as patient engagement facilitators in clinical trials. Clin Ther. 2017;39:2203–2213. doi: 10.1016/j.clinthera.2017.09.014. 29079388

[20] Zhou Q , Ratcliffe SJ , Grady C , Wang T , Mao JJ , Ulrich CM. Cancer clinical trial patient-participants’ perceptions about provider communication and dropout intentions. AJOB Empir Bioeth. 2019;10:190–200. doi: 10.1080/23294515.2019.1618417. 31180295 PMC8653510

[21] Institute of Medicine (US) Forum on Drug Discovery, Development, and Translation. Challenges in Clinical Research [Internet]. Nih.gov. National Academies Press (US); 2010, (https://www.ncbi.nlm.nih.gov/books/NBK50888/) Accessed January 7, 2025.

[22] Wangler J , Jansky M. Primary care involvement in clinical research - prerequisites, motivators, and barriers: results from a study series. Arch Public Health. 2024;82:41–41. doi: 10.1186/s13690-024-01272-x. 38504310 PMC10953082

[23] Sumanen M , Reho T , Heikkilä T , Mäntyselkä P , Halila H , Mattila K. Research orientation among general practitioners compared to other specialties. Scand J Prim Health Care. 2021;39:10–16. doi: 10.1080/02813432.2021.1880072. 33544006 PMC7971219

[24] Gunn J , McCallum Z , Sanci L , Gerner B , Harris C , Wake M. What do GPs get out of participating in research? Experience of the LEAP trial. Aust Fam Physician. 2018;37:372–376. 18464969

[25] Pirotta M , Temple-Smith M. Pracite-based research networks [Internet]. RACGP; 2017. [cited 2025, Jan 7], (https://www.racgp.org.au/getattachment/fb58b0a4-f658-4013-913c-9c32980d4f8d/Practice-based-research-networks.aspx). Accessed January 7, 2025

